# Author Correction: *Drosophila* Alms1 proteins regulate centriolar cartwheel assembly by enabling Plk4-Ana2 amplification loop

**DOI:** 10.1038/s44318-025-00411-6

**Published:** 2025-03-18

**Authors:** Marine Brunet, Joëlle Thomas, Jean-André Lapart, Léo Krüttli, Marine H Laporte, Maria Giovanna Riparbelli, Giuliano Callaini, Bénédicte Durand, Véronique Morel

**Affiliations:** 1 Universite Claude BERNARD Lyon 1, Lyon, France; 2 MeLiS—CNRS-UMR5284, Lyon, France; 3 INSERM-U1314, Lyon, France; 4 Università degli Studi di Siena, Siena, Italy

**Correction to:** *The EMBO Journal* (2025). 10.1038/s44318-025-00382-8 | Published online 28 February 2025

In this article, the grant numbers ANR‐17‐CE13‐0023, 5FI15284XUUK, and DEQ20131029168, which relate to ANR DIVERCIL, Ligue Régionale contre le Cancer, and FRM, respectively, were omitted. The original article has been corrected.

