# Supplementary material for: Drosophila Alms1 proteins regulate centriolar cartwheel assembly by enabling Plk4-Ana2 amplification loop
Source: EMBO J. 2025 Feb 28;44(8):2366–95. doi: 10.1038/s44318-025-00382-8 (PMC12000580; doi:10.1038/s44318-025-00382-8)
Supplement: Supplementary file 1 — Appendix [file 44318_2025_382_MOESM1_ESM.pdf]

Drosophila Alström syndrome proteins regulate centriolar cartwheel assembly by enabling Plk4-Ana2 amplification loop

Marine BRUNET[1,2,3], Joëlle THOMAS[1,2,3], Jean-André LAPART[1,2,3], Léo KRÜTTLI[1,2,3], Marine LAPORTE[1,2,3], Maria Giovanna RIPARBELLI[4], Giuliano CALLAINI[4], Bénédicte DURAND[1,2,3,@,*], Véronique MOREL[1,2,3,@,*].

Appendix Figures

Appendix Figure S1 p.1
Appendix Figure S2 p.3
Appendix Table S1 p.5

Brunet et al.

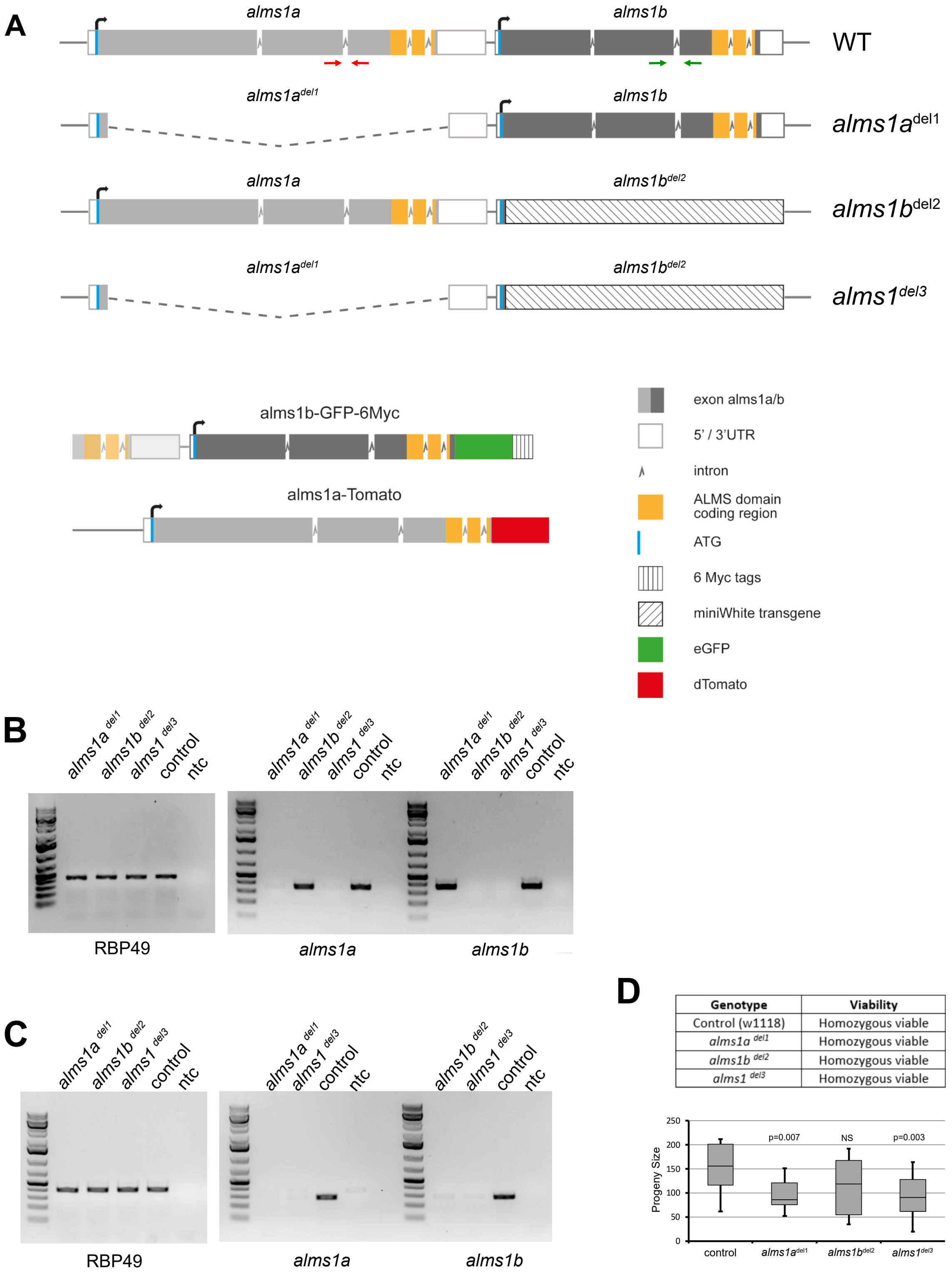

Genotype	Viability
Control (w1118)	Homozygous viable
alms1a del1	Homozygous viable
alms1b del2	Homozygous viable
alms1 del3	Homozygous viable

Appendix Figure S1

Appendix Figure S1: Schemes of *alms1a* and *b* genetic loci and transgenic constructs

(A) Scheme of *alms1a* and *alms1b* locus and genetic tools used in this study. *alms1a* (*CG12179*) and *alms1b* (*CG12184*) are localised on tandem on the minus strand of the X chromosome. The regions coding the ALMS domain are represented in orange. *alms1a*^{del1} was generated by NHEJ with the CRISPR/cas9 strategy. It is a 4270 bp deletion between the position X: 4.635.793, after aa36 in the first exon, and the position X: 4.631.522 in *alms1a* 3'UTR. This deletion removes almost all *alms1a* coding sequence introducing a stop codon at position aa38 of the remaining sequence. *alms1b*^{del2} was generated by HDR with the CRISPR/cas9 strategy. A LoxP-miniWhite-loxP cassette was introduced between the positions X: 4.627.368 (14nt after *alms1b* 3'UTR) and X: 4.630.821 (after aa21), it generated a deletion of 3542 bp, removing almost all *alms1b* coding sequence. *alms1a*^{del1}, *alms1b*^{del2} (*alms1*^{del3}) was generated by HDR with the CRISPR/cas9 strategy on the *alms1a*^{del1} chromosome applying the strategy used to generate *alms1b*^{del2}. For Alms1a-Tomato transgene, the genomic sequences including *alms1a* transcribed sequence and 1737 bp upstream of the ATG were used. dTomato coding sequence was inserted in frame after the last coding exon of *alms1a*. The transgene was inserted by targeted mutagenesis on the chromosome III at the 89E11 locus. For Alms1b-GFP transgene, the genomic sequences including *alms1b* transcribed sequence and 1505 bp upstream of the ATG (which include the 3 last exons of *alms1a*) were used. A 9aa linker, the eGFP coding sequence and 6 Myc tags were inserted in frame after the last coding exon of *alms1b*. The transgene was inserted by targeted mutagenesis on the chromosome II at the 53B2 locus. (B) Validation PCR of *alms1* deletions on genomic DNA. Primer couples specific of *alms1a* and *b* are depicted on WT chromosome scheme respectively in red and green. Left, *rpb49* control gene, middle, *alms1a*, *alms1a* is only detected in *alms1b* ^{del2} and control, right, *alms1b*, *alms1b* is only detected in *alms1a* ^{del1} and control. (C) *alms1* deletions are RNA null alleles. Validation PCR (using the same primers as above) performed on cDNA obtained from *alms1a* ^{del1}, *alms1b* ^{del2}, *alms1*^{del3} and control testes. Left, *rpb49* control gene; middle, *alms1a* cDNA is only detected in control but neither in *alms1a* ^{del1} nor in the double *alms1a, alms1b* deletion (*alms1*^{del3}); right, *alms1b* cDNA is only detected in control but neither in *alms1b* ^{del2} nor in the double *alms1a, alms1b* deletion (*alms1*^{del3}). (D) Table summarizing viability of *alms1a*^{del1}, *alms1b*^{del2} and *alms1*^{del3} fly lines. Progeny size of fertile males for each genotype and w^{1118} control line (control: n=29 males, *alms1a*^{del1}: n=16, *alms1b*^{del2}: n=25 and *alms1*^{del3}: n=27). p-values of the Wilcoxon test comparing control to the deletion lines are given on the graph.

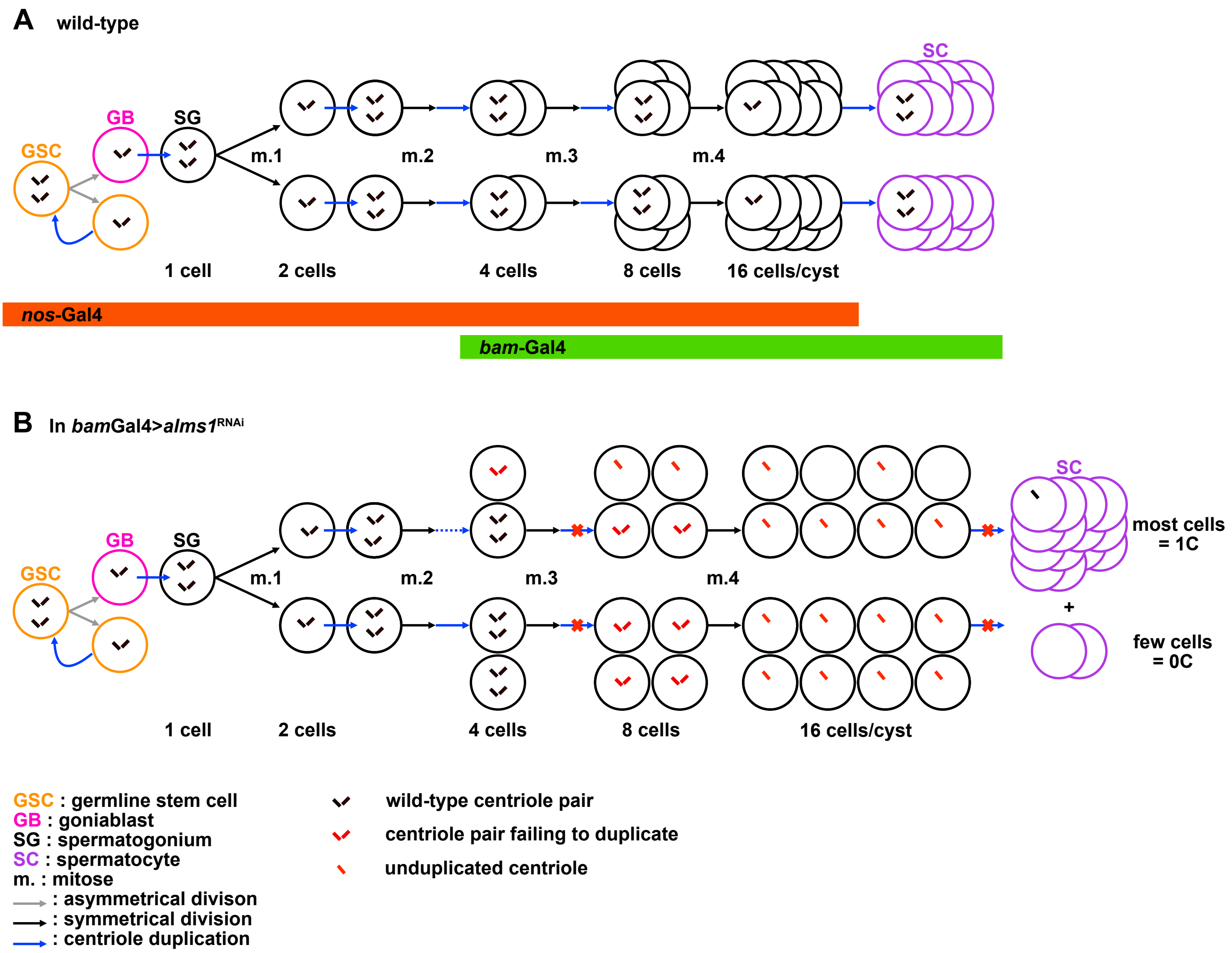

Appendix Figure S2

Appendix Figure S2. Centriole duplication events during *Drosophila* spermatogenesis

Schemes representing centriole duplication events during spermatogenesis progression in (A) *bam*-Gal4>*lacZ*^{RNAi} (control condition) or (B) *bam*-Gal4>*alms1*^{RNAi}. Spermatogenesis starts with the asymmetric division (grey arrow) of a germline stem cell (GSC, orange) which gives rise to a GSC and a gonialblast (GB, pink) that initiates differentiation into a spermatogonium (SG, black) while it duplicates its centrioles (blue arrow). SG undergo four symmetric divisions (black arrow, mitoses m.1-4) to generate a cyst of 16 cells. Prior to each mitosis, centrioles duplicated resulting in cells with 4 centrioles arranged in two pairs. After mitosis 4, SGs complete a pre-meiotic S phase and duplicate their centrioles while they differentiate into spermatocytes (SCs, purple).

bam-Gal4 induces the expression of the *alms1*^{RNAi} (red cross) in spermatogonial cysts, and thus centriole duplication failure in some cells within 4-cells spermatogonial cysts and all cells in 8-cells cysts (centrioles in cells affected are red).

EMBOJ-2024-118083-T

name	sequence 5'-3'
F-Alms1a-Tom	TAATTC**AGATCT**TCTCCATGACCAAGGAGACC
R-Alms1a-Tom	TAATTC**GCGGCCGC**AGCATGATTGCCATGCTGTGAT
F-Alms1b-GFP	ATTGGGAATTCGTTAAC**AGATCT**ACCACTCATCAGTACGATG
R-Alms1b-GFP	CGAGCCGCGGCCGC**AGATCT**TCGATCTTCTCTAGATGATTAC
F-5'UTR-V5-Alms1a	CGGGGATCAGATCC**GCGGCCGC**TCAATTTGTTTTCGAATGATTTT
R-5'UTR-V5-Alms1a	ACGTTCGAGGTCGAC**TCTAGA**TGGTGCTATCCAGGCCCAGCAGGGGGTTGGGGATGGGCTTGCCCATGGTCGAAAAAGTTTATTCCCCT
F-CDSAlms1a	GCCTGGATAGCACCATGAGAGCGAAGAGAGGAGC
R-CDSAlms1a	CGTTCGAGGTCGAC**TCTAGA**TATTTTTCTCAAATTTTTATTAGTCTTGTAAATTTC
gRNA1Alsm1a	CGATAACTTTCGATATCAGG
gRNA2Alsm1a	GGACTAAACCATACGTGCTT
gRNA3Alsm1a	CAATTTGTTATGGCGAGAAC
gRNA4Alms1a	GATTGCCATGCTGTGATTCA
gRNA5Alms1a	CAGGGAATATATCAGCGCAA
gRNA6Alms1a	GATATCCCTGAATCACAGCA
F-Alsm1aKO	GAACCGGTTCTCAATAGGC
R-Alsm1aKO	GAACCGGTTCTCAATAGGC
F2-Alms1a	CCAGCAGCAGCAACAGTTTG
R2-Alms1a	GCTACCACTCTGGTGTGGAG
F-5’armAlms1b	TAATTC**GCTAGC**GATGAATGCAGAGAGTATGC
R-5’armAlms1b	TAATTC**GGTACC**TGGTGGATGAACGCGGGT
F-3’armAlms1b	TAATTC**AGATCT**GTGTGGCCAAATGCCTAT
R-3’armAlms1b	TAATTC**CCTAGG**CAACAAGTGAACGCAACTCG
gRNA1Alsm1b	ATATAATAATCCCCGGGTGG
gRNA2Alsm1b	TTTAAATCTATATCGCAGTG
F-5'Alsm1bKO	ATACAAACTCTTCAAGCTGC
R-5'Alsm1bKO	TCTTTTCGCGAACATTCGAGG
F1-3'Alsm1bKO	ATATCTGGCTCTAAGACTTCG
R-3'Alsm1bKO	CGCCAACAACAACAATAGC
F-5’armAlms1aΔ	TAATTC**GCTAGC**GGGTTGTTGCTGCGGATTCG
F2-5'Alsm1bKO	CGGGCAGCTCCAAATTCAGG
F2-3'Alsm1bKO	CGTGTTTACTGTTTATTGC
F3-Alms1b	TTGTGGTCAGCGGACTTACG
R3-Alms1b	CTCACATCTGGTGATGGACG
F-RBP49	AAGATCGTGAAGAAGCGCAC
R-RBP49	ACTCGTTCTCTTGAGAACGC

Bold letters represent cloning restriction sites

Appendix Table S1 List of Primers used in the study
