# Supplementary figures and images for: Drosophila Alms1 proteins regulate centriolar cartwheel assembly by enabling Plk4-Ana2 amplification loop
Source: EMBO J. 2025 Feb 28;44(8):2366–95. doi: 10.1038/s44318-025-00382-8 (PMC12000580; doi:10.1038/s44318-025-00382-8)

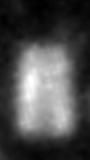

Supplement: Supplementary file 3 — Source data Fig. 2 [file 44318_2025_382_MOESM3_ESM.zip › Fig_2/A/RPE1_Tubulin(594)_ALMS1(647)_SIDE.tif]

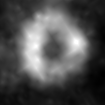

Supplement: Supplementary file 3 — Source data Fig. 2 [file 44318_2025_382_MOESM3_ESM.zip › Fig_2/B/RPE1_Tubulin(594)_ALMS1(647)_TOP.tif]

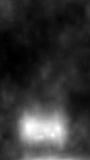

Supplement: Supplementary file 3 — Source data Fig. 2 [file 44318_2025_382_MOESM3_ESM.zip › Fig_2/C/1_RPE1_Tubulin(594)_ALMS1(647).tif]

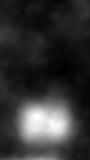

Supplement: Supplementary file 3 — Source data Fig. 2 [file 44318_2025_382_MOESM3_ESM.zip › Fig_2/C/2_RPE1_Tubulin(594)_ALMS1(647).tif]

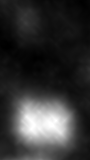

Supplement: Supplementary file 3 — Source data Fig. 2 [file 44318_2025_382_MOESM3_ESM.zip › Fig_2/C/3_RPE1_Tubulin(594)_ALMS1(647).tif]

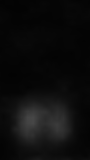

Supplement: Supplementary file 3 — Source data Fig. 2 [file 44318_2025_382_MOESM3_ESM.zip › Fig_2/C/4_RPE1_Tubulin(594)_ALMS1(647).tif]

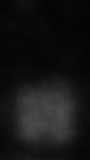

Supplement: Supplementary file 3 — Source data Fig. 2 [file 44318_2025_382_MOESM3_ESM.zip › Fig_2/C/5_RPE1_Tubulin(594)_ALMS1(647).tif]

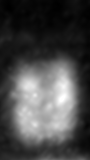

Supplement: Supplementary file 3 — Source data Fig. 2 [file 44318_2025_382_MOESM3_ESM.zip › Fig_2/C/6_RPE1_Tubulin(594)_ALMS1(647).tif]

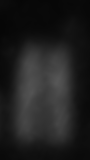

Supplement: Supplementary file 3 — Source data Fig. 2 [file 44318_2025_382_MOESM3_ESM.zip › Fig_2/C/7_RPE1_Tubulin(594)_ALMS1(647).tif]

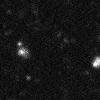

Supplement: Supplementary file 9 — Source data Fig. 6C [file 44318_2025_382_MOESM9_ESM.zip › Fig6C-raw/240321_Alms1a_T3_2_2C-1.tif]

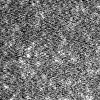

Supplement: Supplementary file 9 — Source data Fig. 6C [file 44318_2025_382_MOESM9_ESM.zip › Fig6C-raw/240503_Alms1a+Plk4_T2_2_2C-3.tif]

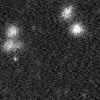

Supplement: Supplementary file 9 — Source data Fig. 6C [file 44318_2025_382_MOESM9_ESM.zip › Fig6C-raw/240503_Alms1a+Plk4_T2_2_3C-1.tif]

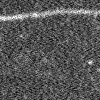

Supplement: Supplementary file 9 — Source data Fig. 6C [file 44318_2025_382_MOESM9_ESM.zip › Fig6C-raw/240503_Alms1a+Plk4_T2_3_3+C-6.tif]

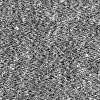

Supplement: Supplementary file 9 — Source data Fig. 6C [file 44318_2025_382_MOESM9_ESM.zip › Fig6C-raw/240503_Plk4_T1_2_3+C-4.tif]

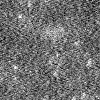

Supplement: Supplementary file 9 — Source data Fig. 6C [file 44318_2025_382_MOESM9_ESM.zip › Fig6C-raw/240503_Plk4_T2_2_2C-1.tif]

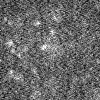

Supplement: Supplementary file 9 — Source data Fig. 6C [file 44318_2025_382_MOESM9_ESM.zip › Fig6C-raw/240503_Plk4_T2_2_3C-4.tif]
