# Supplementary material for: Drosophila Alms1 proteins regulate centriolar cartwheel assembly by enabling Plk4-Ana2 amplification loop
Source: EMBO J. 2025 Feb 28;44(8):2366–95. doi: 10.1038/s44318-025-00382-8 (PMC12000580; doi:10.1038/s44318-025-00382-8)
Supplement: Supplementary file 11 — Expanded View Figures [file 44318_2025_382_MOESM11_ESM.pdf]

Expanded View Figures

Figure EV1. Alms1a and Alms1b dynamics of localisation.

(**A**) Unexpended images of Alms1b-GFP (magenta) localisation prior to spermatids formation, during the second meiotic division. The two newly separated centrioles display Alms1b at their proximal end. Ana1-Tomato (green) as a centriolar marker. (**B**) Dynamics of localisation of endogenous Alms1a (top row, red) and endogenous Alms1b (bottom row, magenta) from spermatogonia to round spermatid stages. As observed with Alms1a-Tom transgene, endogenous Alms1a is detected from the spermatogonia until after the round spermatid stages at the proximal side of centrioles (labelled in green with Asl). As for Alms1b-GFP, endogenous Alms1b is not detected in spermatogonia. It is first observed in round spermatids in contrast to Alms1b-GFP which is detected from the late SC stage. This difference in timing could be due to technical difficulties to observe minute amounts of proteins with the antibody compared to the GFP-tagged protein. Endogenous proteins thus show dynamics of localisation at centrioles similar to that described with Alms1a-Tom and Alms1b-GFP. (**C**) Alms1b-GFP (magenta) localisation in neuroblasts (NB, dotted white outline) and ganglion mother cell (GMC, plain white outline). We did not detect Alms1b-GFP in NB, GMC or any other larval brain cell type. Miranda-mCherry (yellow) used as a landmark for GMC and NB. Nuclei in grey. (**D**) Alms1b-GFP (magenta) localisation in syncytial embryo (top) and cellularised embryo (bottom). No Alms1b-GFP is detected during the syncytial stage. Alms1b-GFP starts to localise at spindle pole during the last synchronous mitosis (cycle 14) in cellularised embryos Tubulin in green, DNA in cyan. Scale bars: (**A**, **C**, **D**) 5 µm, (**A**, inset, **B**) 1 µm.

© The Author(s)

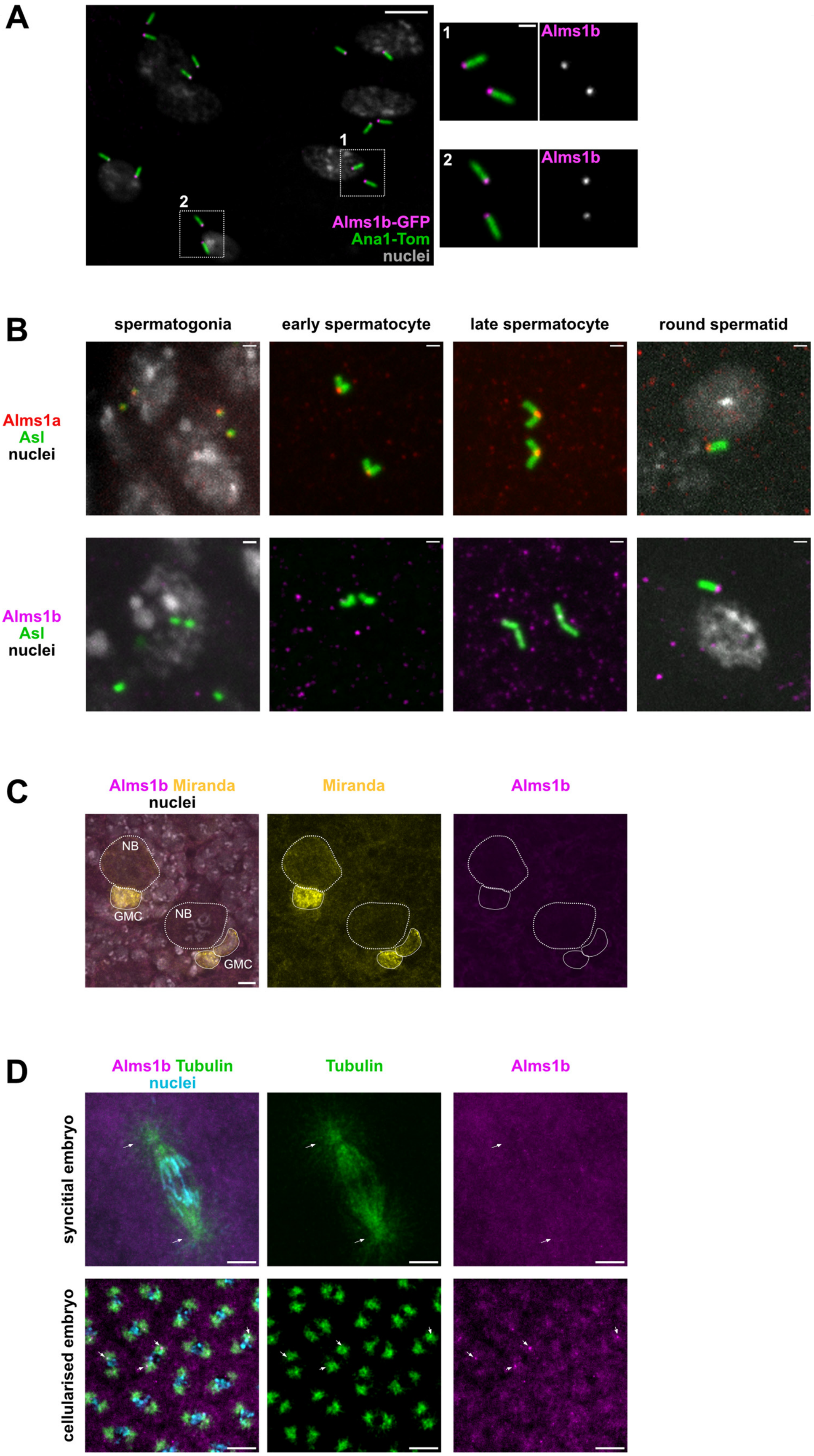
A
1
2
Alms1b-GFP
Ana1-Tom
nuclei
Alms1b
Alms1b
B
spermatogonia
early spermatocyte
late spermatocyte
round spermatid
Alms1a
Asl
nuclei
Alms1b
Asl
nuclei
C
Alms1b Miranda
nuclei
Miranda
Alms1b
NB
GMC
NB
GMC
D
Alms1b Tubulin
nuclei
Tubulin
Alms1b
syncitial embryo
cellularised embryo

 © The Author(s)

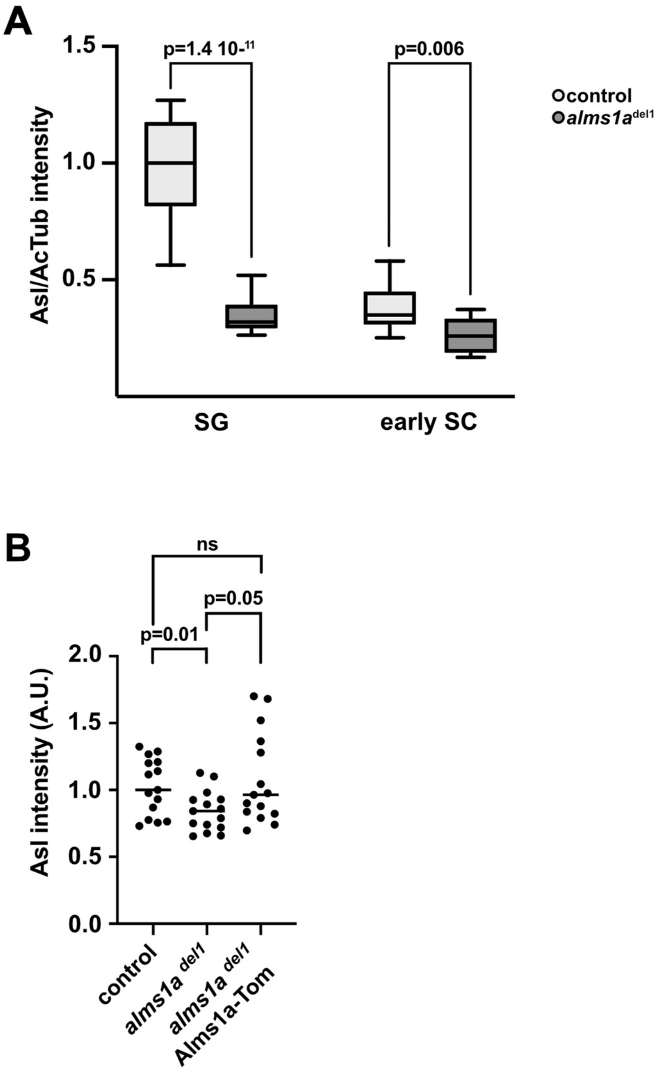

Figure EV2. Asl recruitment in impaired upon loss of *alms1a*.

(**A**) Comparison of Asl intensity at centrosomes normalised to acetylated α-tubulin intensity in U-ExM control and *alms1a*^{del1} testis. Acetylated α-tubulin labels the centriole and was used as an internal reference for Asl intensities comparison between samples. Control: $n = 3$ testis, SG = 20 centrosomes, early SC = 21 centrosomes; *alms1a*^{del1}: $n = 3$ testis, SG = 12 centrosomes, early SC = 20 centrosomes. The Box Plots show the interquartile range (IQR), with the median (50th percentile) indicated by the horizontal line inside the box and the whiskers extending to the 10th and 90th percentiles. (**B**) Comparison of Asl intensity at centrosomes of early SC of control, *alms1a*^{del1} and *alms1a*^{del1};;Alms1a-Tomato testes. Reduction of Asl intensity in *alms1a*^{del1} is rescued to control levels upon introduction of Alms1a-Tomato transgene. Analysis based on immuno-fluorescence with anti Asl antibody. control: 3 testes, 15 centrosomes; *alms1a*^{del1}: 2 testes, $n = 15$; *alms1a*^{del1};;Alms1a-Tomato: 3 testes, $n = 15$. P values were obtained with two-sided unpaired Wilcoxon test. Source data are available online for this figure.

© The Author(s)

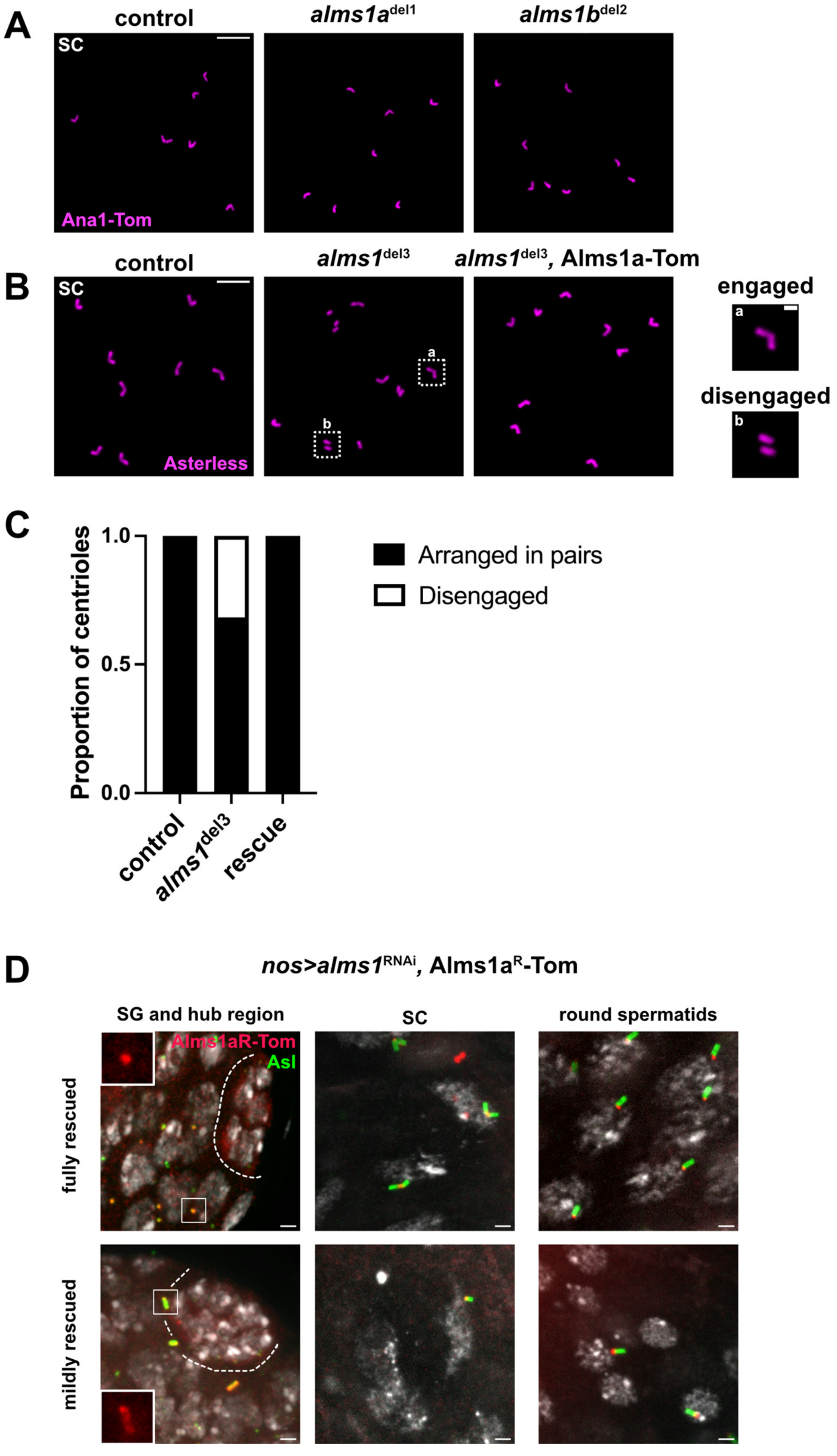
A
control
alms1a del1
alms1b del2
SC
Ana1-Tom
B
control
alms1 del3
alms1 del3, Alms1a-Tom
engaged
disengaged
SC
Asterless
a
b
C
Proportion of centrioles
1.0
0.5
0.0
Arranged in pairs
Disengaged
control
alms1 del3
rescue
D
nos>alms1 RNAi, Alms1a R-Tom
SG and hub region
SC
round spermatids
fully rescued
mildly rescued
Alms1aR-Tom
Asl

 © The Author(s)

◀ **Figure EV3. Phenotypes of *alms1* mutants.**

Unexpended images of SC showing centrioles in (**A**) *alms1a*^{del1} (middle), *alms1b*^{del2} (right) and in *alms1b*^{del2} rescued by Alms1b-GFP as a control (left, similar behaviour is observed for *alms1a*^{del1} rescued by Alms1a-Tom). Ana1-Tomato (magenta) as a centriolar marker. (**B**) Centrioles in control (left), *alms1*^{del3} (middle) and *alms1*^{del3};;Alms1a-Tom (right). For *alms1*^{del3}, a focus on engaged (a) and disengaged (b) centrioles is shown below. Asl (magenta) as a centriolar marker. (**C**) Quantification of centriole disjunction in control, *alms1*^{del3} and *alms1*^{del3};;Alms1a-Tom testes. The double *alms1a,b* deletion (*alms1*^{del3}) results in defective centriole cohesion for 26% of centrioles ($n = 865$ centrioles, 4 males). Centriole cohesion defect is fully rescued by expression of Alms1a-Tom (*alms1*^{del3};;Alms1a-Tom) with none of the centriole pair observed presenting centriole cohesion defects (n = 666 centrioles, 3 males), as also observed in a wild-type line (100% centrioles in pairs, $n = 454$ centrioles, 3 males). (**D**) Partial rescue of the centriole duplication defect upon introduction in *nos*-Gal4>*alms1*^{RNAi} background of the Alms1a^{R}-Tom transgene. The Alms1a^{R}-Tom is a modified Alms1a-Tom that carries silent mutations aiming at providing resistance against *alms1*^{RNAi} mediated degradation (this study). 41% (11/27) of *nos*-Gal4>*alms1*^{RNAi};;Alms1a^{R}-Tom testes did not show any rescue of the duplication defects. We however observed either mild or almost complete rescue of centriole duplication in respectively 48% (13/27) and 11% (3/27) of the observed testes. Bottom row: in mildly rescued testes few centrioles (marked with Asl, green) were observed in GSCs (cells contacting the hub, delineated with a white dotted line). When centrioles could be observed they were longer than in control conditions and were decorated by Alms1a^{R}-Tom (red) on their full length (Alms1a^{R}-Tom fluorescence of the boxed centriole is shown at the bottom left corner). SC (middle image) contained either no or only single centrioles and only a subset of round spermatids had a centriole (right). Top row: in fully rescued testes, centrioles co-labelled with Asl and Alms1a^{R}-Tom were observed in GSCs (left image, inset shows Alms1a^{R}-Tom localisation at the boxed centriole) and SG. SC contained pairs of centrioles (middle row) and most round spermatids contained a centriole (right). In both mildly and fully rescued testes, observed centrioles showed Alms1a^{R}-Tom localisation at their proximal end, as observed in control conditions. The partial rescue achieved likely reflects an incomplete resistance of the Alms1a^{R}-Tom construct to *alms1*^{RNAi} mediated degradation. Occurrence of rescue events by Alms1a-Tom nevertheless shows the specificity of the *alms1* RNAi construct used. Centrioles labelled with anti Asl (green), Alms1a^{R}-Tom (red). Scale bars: (**A**, **B**) 5 μm, (**B**, insets) 1 μm, (**D**) 2 μm. Source data are available online for this figure.

© The Author(s)

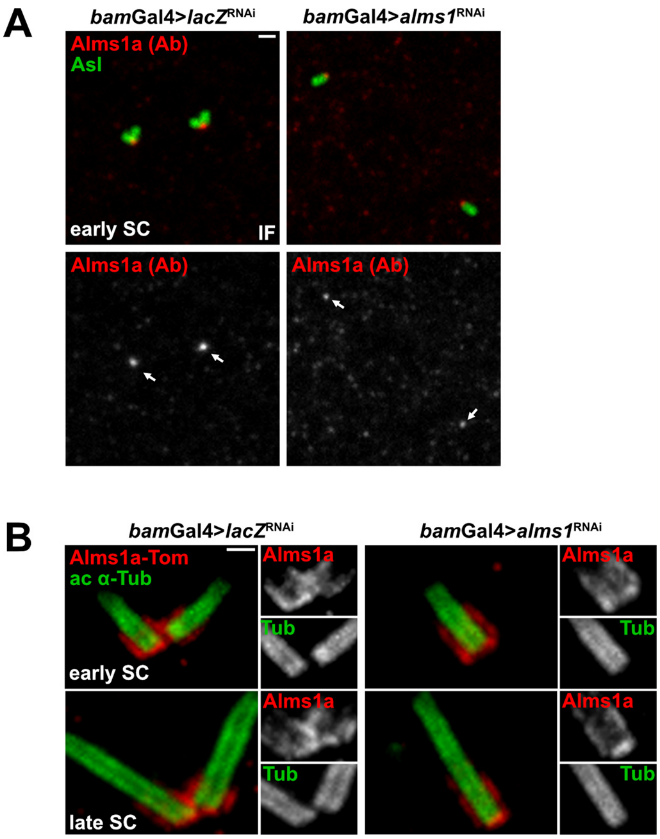

Figure EV4. Alms1a localises at the proximal side of the remaining centriole in *bam*-Gal4>*alms1*RNAi.

(**A**) Immunofluorescence images of *bam*-Gal4>*lacZ*RNAi and *bam*-Gal4>*alms1*RNAi stained with anti-Alms1a (red) and anti Asl (green) antibodies showing endogenous Alms1a localisation at the proximal end of the centrioles. (**B**) U-ExM images of Alms1a-Tomato (red) in *bam*-Gal4>*lacZ*RNAi (*bam*-Gal4>*lacZ*RNAi;;Alms1a-Tom) or *bam*-Gal4>*alms1*RNAi centrioles (*bam*-Gal4>*alms1*RNAi;;Alms1a-Tom). Centriolar walls are revealed with acetylated α-tubulin (green, ac α-tub). Alms1a-Tom forms a cup surrounding the proximal extremity of both mother and daughter centrioles in *bam*-Gal4>*lacZ*RNAi as well as the proximal side of the unduplicated centriole in *bam*-Gal4>*alms1*RNAi. Scale bars: (**A**) 1 µm, (**B**), after expansion factor correction, 250 nm.

 © The Author(s)

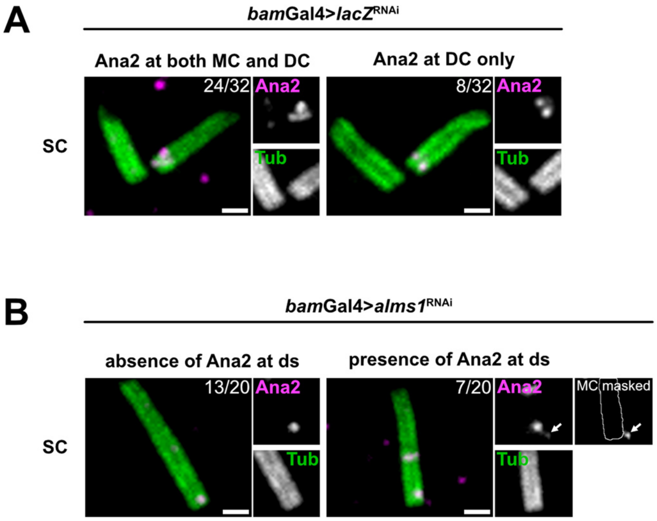

Figure EV5. Ana2 centriolar localisation in spermatocytes.

U-ExM images of Ana2-mNeonGreen knock-in (Ana2-eNG, in magenta) in (**A**) *bam*-Gal4>*lacZ*^{RNAi} or (**B**) *bam*-Gal4>*alms1*^{RNAi} showing Ana2 localisation at centrioles in spermatocyte stage (SC). Centriolar walls are revealed with acetylated α-tubulin (green, ac α-tub), Mother centriole (MC, on the left of *bam*-Gal4>*lacZ*^{RNAi} images). Scale bars (after expansion factor correction): 250 nm.

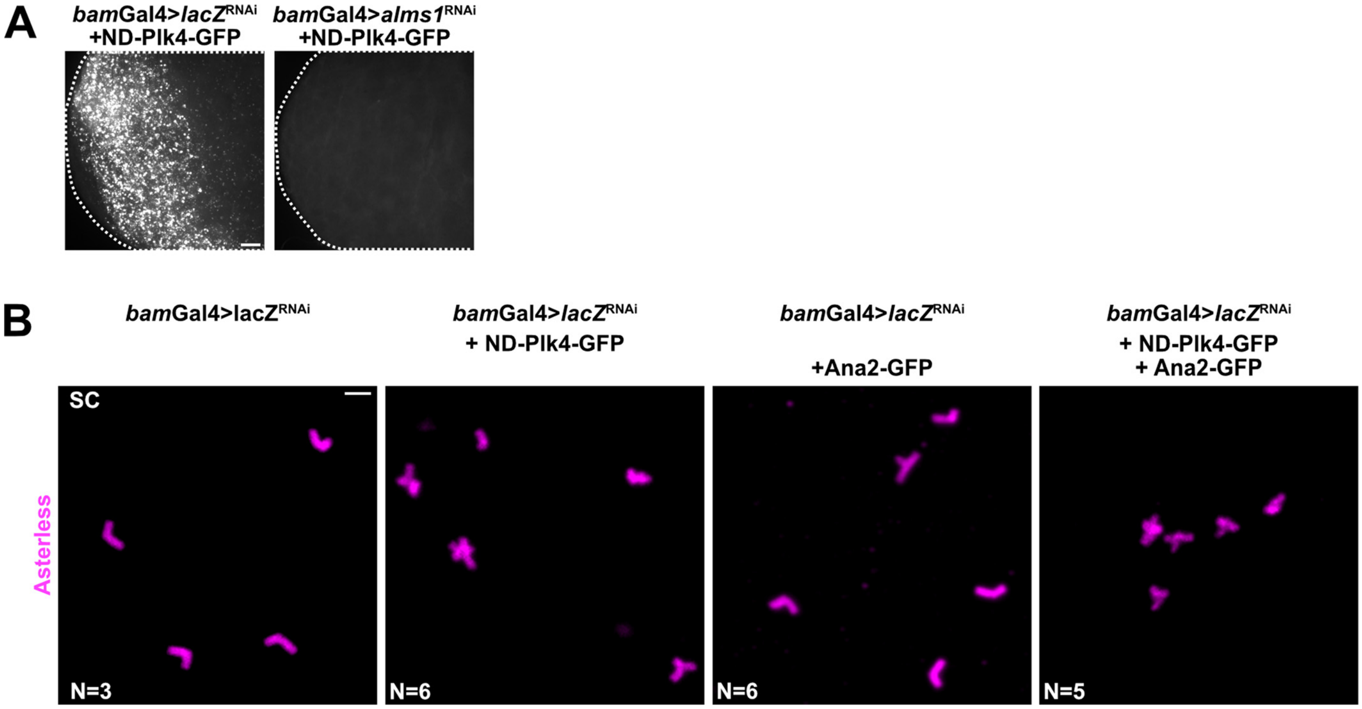

Figure EV6. Overexpression of ND-Plk4-GFP in *bam*-Gal4>*lacZ*^{RNAi} and *bam*-Gal4>*alms1*^{RNAi} testes.

(**A**) Images of unexpanded *bam*-Gal4 > {*lacZ*^{RNAi}, ND-Plk4-GFP} or *bam*-Gal4 > {*alms1*^{RNAi}, ND-Plk4-GFP testis hub regions. In control condition, ND-Plk4-GFP (grey) forms aggregates in the cytoplasm while we only observe diffuse ND-Plk4-GFP in *alms1*^{RNAi}. The hub region is outlined with a dotted line. (**B**) Representative immunofluorescence images of centrosomes with or without centriole overduplication upon overexpression of ND-Plk4-GFP, Ana2-GFP or both in *bam*-Gal4>*lacZ*^{RNAi} genetic background. Following overexpression of ND-Plk4-GFP most centrosomes presented overduplication of centrioles including numerous events of centrosomes with more than 3 centrioles. In contrast, overexpression of Ana2-GFP induces the formation of few centrosomes with three centrioles while rosettes (> 3C) were never observed. Co-expression of ND-Plk4-GFP and Ana2-GFP leads to massive overduplication of centrioles, to an extent at least as important than observed with ND-Plk4-GFP alone. Centrioles labelled with anti Asl antibody (magenta). Scale bars: (**A**) 10 µm, (**B**) 1 µm.

 © The Author(s)
