# Supplementary material for: Drosophila Alms1 proteins regulate centriolar cartwheel assembly by enabling Plk4-Ana2 amplification loop
Source: EMBO J. 2025 Feb 28;44(8):2366–95. doi: 10.1038/s44318-025-00382-8 (PMC12000580; doi:10.1038/s44318-025-00382-8)
Supplement: Supplementary file 10 — EV and Appendix Figure Source Data [file 44318_2025_382_MOESM10_ESM.zip › EMBOJ-2024-118083R1-Figure_S1_Source_Data-sd.zip!/Fig_S1/S1A/alms scheme.docx]

**Figure S1: Schematic representations of *alms1a* and *b* loci and genetic constructions**

*alms1a* (CG12179) and *alms1b* (CG12184) schematic representation at scale. The genes are localized on tandem on the minus strand of the X chromosome. The regions coding the ALMS domain are represented in orange.

*alms1a ^Δ^* was generated by NHEJ with the CRISPR/cas9 strategy. It is a 4270 nucleotides (nt) deletion between the position X: 4.635.793, after aa36 in the first exon, and the position X: 4.631.522 in *alms1a* 3’UTR. This deletion thus removes almost all *alms1a* coding sequence. In addition, the 5’ break point introduces a stop codon at position aa38.

*alms1b ^Δ^* was generated by HDR with the CRISPR/cas9 strategy. A LoxP-miniWhite-loxP cassette (ref) was introduced between the positions and X: 4.627.368 (14 nt after *alms1b* 3’UTR) and X: 4.630.821 (after aa21), it generated a deletion of 3542 nucleotides, removing almost all *alms1b* coding sequence.

*alms1a ^Δ^*, *alms1b ^Δ^* (*alms1 ^2Δ^*) was generated by HDR with the CRISPR/cas9 strategy on the *alms1a ^Δ^* chromosome applying the strategy used to generate *alms1b ^Δ^*.

For Alms1a-Tomato transgene, the genomic sequences including *alms1a* gene and the 890nt nucleotide upstream of the gene and containing *alms1a* promoter, were used. dTomato coding sequence was inserted in frame after the last coding exon of *alms1a.* The transgene was inserted by targeted mutagenesis on the chromosome III at the 89E11 locus.

For Alms1b-GFP transgene, the genomic sequences including *alms1b* gene and the 1452nt nucleotides upstream of the gene (which include the 3 last exons of *alms1a*) and containing *alms1b* promoter, were used. A 9 aa linker, the eGFP coding sequence and 6 Myc tags were inserted in frame after the last coding exon of *alms1b.* The transgene was inserted by targeted mutagenesis on the chromosome II at the 53B2 locus.
